# Functions of the C2H2 Transcription Factor Gene thmea1 in* Trichoderma harzianum* under Copper Stress Based on Transcriptome Analysis

**DOI:** 10.1155/2018/8149682

**Published:** 2018-07-18

**Authors:** Jie Mei, Lirong Wang, Xiliang Jiang, Beilei Wu, Mei Li

**Affiliations:** Institute of Plant Protection, Chinese Academy of Agricultural Sciences, Beijing 100081, China

## Abstract

*Trichoderma* spp. are important biocontrol filamentous fungi and have tremendous potential in soil bioremediation. In our previous studies, a C2H2 type transcription factor coding gene (*thmea1*) was cloned from a biocontrol agent* T. harzianum* Th-33; the encoded sequence of* thmea1* contained 3 conserved C2H2 domains with Swi5 and Ace2 in* Saccharomyces cerevisiae*. The* thmea1* knockout mutant* Δthmea1* showed 12.9% higher copper tolerance than the wild-type Th33. To elucidate the function of* thmea1* and its relationship with copper stress response, we conducted transcriptome sequencing and analysis of wild-type Th33 and* Δthmea1* under 0.8 mM copper stress. A total of 1061 differentially expressed genes (DEGs) were identified between the two strains, all DEGs were assigned to KEGG pathway database, 383 DEGs were annotated in 191 individual pathways, and the categories of ribosomal protein synthesis and amino acid metabolism were the most highly enriched ones. Analysis of related DEGs showed that the expression levels of intracellular glutathione detoxification enzyme, heat shock proteins, and ribosomal proteins in* Δthmea1* were higher than that of the wild-type Th33, and the expression of metallothionein (MT) gene did not change. In addition, the expression levels of genes coding for proteins associated with the Ccc2p-mediated copper chaperone Atx1p transport of copper ions into the Golgi secretory pathway increased, as well as the copper amine oxidase (CuAO). These findings suggest that Thmea1 is a negative regulated factor of copper tolerance ability in* T. harzianum*. It does not show metallothionein expression activator activities as that of Ace2 in* S. cerevisiae*. We hypothesize that after* T. harzianum* has lost its* thmea1* gene, the ability of cells to scavenge reactive oxygen species, mainly through the glutathione antioxidant system, is enhanced, whereas protein synthesis and repair and copper secretion increase under copper stress, which increases the ability of the mutant strain to tolerate copper stress.

## 1. Introduction

*Trichoderma* spp. are important biocontrol filamentous fungi which are widely used to prevent soil-borne diseases in plants as active ingredients in biofertilizers and biopesticides. In addition,* Trichoderma* spp. also have tremendous potential in soil bioremediation [1–3]. In recent years, copper and copper-containing compounds have been extensively utilized in the preparation of microbicides and chemical pesticides and as livestock food additives, consequently resulting in a continuous increase of soil copper levels [4, 5]. Researches on copper stress responses and metabolism mechanisms in* Trichoderma* species have facilitated the improvement of copper tolerance as well as the prevention and treatment of plant diseases.

Transcription factors (TFs), also known as* trans*-acting factors, are proteins that can directly or indirectly interact with* cis*-acting elements in gene promoters to regulate the start of gene transcription [6]. The* thmea1* gene of* T. harzianum* Th33 was cloned and was 1,446 bp in length. It did not contain any intron and the encoded sequence contained three conserved C2H2 zinc finger domains that were identical to the yeast activator of HO gene transcription Swi5 and metallothionein expression activator Ace2p.* thmea1* was speculated to be a C2H2-type transcription factor gene (GenBank Accession Number MF802279). A knockout mutant of this gene,* Δthmea1*, was obtained using a homologous double-crossover method. Studies have shown that when* Δthmea1 *was growing on potato dextrose agar (PDA) culture media containing 0–2.4 mM copper ions, its growth rate significantly increased compared to the wild-type strain Th33, and its median inhibitory concentration (MIC_50_) to copper ions was 1.92 mM, which was 12.9% higher than that of the wild-type strain. These findings showed that* thmea1* was associated with copper tolerance in* Trichoderma* and might participate in copper stress responses and copper metabolism. To further study the function of this gene, we conducted transcriptome sequencing of wild-type Th33 and* Δthmea1* under 0.8 mM copper treatment, analyzed DEGs, and examined the function of the* thmea1* gene in this study. The findings of the present study provide a foundation for the elucidation of copper stress responses and metabolism mechanisms in* Trichoderma* species.

## 2. Materials and Methods

### 2.1. Test Strains

The wild-type* T. harzianum* Th-33 was isolated from soil samples in the Beijing region as described previously [7]. The* thmea1 *knockout mutant* Δthmea1* was created with hygromycin B resistance by homologous recombination and then purified by isolation of single conidia and was stored in our laboratory.

### 2.2. Determination of* Trichoderma* Growth Rate

*T. harzianum* Th33 wild-type strain and the mutant* Δthmea1* were inoculated on the PDA media for activation for three days at 28°C. Then, a culture disc was removed with a cork borer (5-mm diameter) and inoculated on the center of PDA plates with copper iron concentrations of 0 mM, 0.8 mM, 1.6 mM, 2.4 mM, 3.2 mM, and 4.0 mM. Quadruplicates were prepared for each concentration, and the plates were grown at 28°C. The growth status of the fungi was observed at 24-h intervals, and the diameter of the fungal colonies was recorded. The MIC_50_ of* T. harzianum* to copper ions was calculated as described elsewhere [8]. A standard curve of the colony growth inhibition rate to its corresponding copper concentration was constructed, and inhibition rate was calculated using the following equation: Inhibition rate (%) = [(Diameter of control colony (cm) − Diameter of treated colony (cm)]/Diameter of control colony (cm) × 100%. The copper concentration at a growth inhibition rate of 50% was calculated using a regression formula, which is the MIC_50_.

### 2.3. Strain Treatment and Sampling

Approximately 100 *μ*L of Th33 and* Δthmea1* spore suspensions at a density of 1 × 10^7^/mL were inoculated into 250-mL conical flasks containing 100 mL potato dextrose (PD) liquid culture media. Each strain was inoculated in duplicate. The flasks were cultured in a shaking incubator at 28°C and 180 rpm for 39 h. This was followed by the addition of 1 mL of filter-sterilized copper sulfate solution (80 mM) to a final copper concentration of 0.8 mM. The flasks were then cultured for another 4 h. The above fermentation broth samples were filtered and washed thrice to collect the hyphae. Aliquots of the samples were then prepared, flash frozen in liquid nitrogen, and then stored at −80°C.

### 2.4. Total RNA Extraction

Total RNA was extracted using Trizol reagent (Invitrogen, CA, USA) according to the manufacturer's instruction. The RNA quality and quantity were determined using an Agilent 2100 Bioanalyzer. The Qubit RNA Assay Kit was used for accurate quantification of the initial total RNA.

### 2.5. Construction of cDNA Libraries and Sequencing

We commissioned Berry Genomics Co., Ltd., to conduct transcriptome sequencing using the Illumina HiSeq 2500 platform. Dynabeads mRNA DIRECT kits (Invitrogen, CA, USA) were used to extract mRNA from total RNA. After mRNA fragmentation, random primers were used to synthesize the first-strand cDNA through reverse transcription before synthesis of the second-strand cDNA to obtain double-stranded cDNA. End-repair and addition of adenines to the 3′ terminus of the double-stranded cDNA was performed, followed by ligation of sequencing adapters. After purification of the ligated products, PCR amplification was conducted, and the PCR products were separated using 2% agarose gel electrophoresis. The target 400–500 bp band was extracted from the gel and recovered as the final library. qPCR was used for quality control of the libraries prior to loading on the machine for sequencing.

### 2.6. Gene Annotation and Quantitative Analysis of Expression Levels

Tophat software was used for alignment and annotation of the reads from the sequenced samples and* T. harzianum* Th33 genome data (GenBank Acc. No. PRJNA272949) [7]. Gene expression levels were expressed as fragments per kilobase of transcript per million fragments mapped (FPKM) [9], i.e., the number of matches for every kilobase of transcript per million fragments.

### 2.7. Analysis of Differentially Expressed Genes (DEGs)

DEGs between samples were generated using Cuffdiff, in which the fold-change was the ratio of the expression levels between the two samples; i.e., log_2_(FC) was log_2_ (fold-change) = log_2_(Sample A/Sample B). Here, we set p value≤0.01 and ∣log_2_(FC)∣≥1 as significant differences. The DEGs were identified and functionally annotated using the databases of Gene Ontology (GO) and KEGG.

### 2.8. Validation of Transcriptome Sequencing Results

To confirm the reliability of transcriptome sequencing results, we selected 12 DEGs for real-time fluorescence quantitative PCR (qRT-PCR) validation. qRT-PCR was conducted using the SYBR Green reagent kit (SuperReal PreMix Plus SYBR Green kit FP205, Tiangen). Each 20-*μ*L PCR reaction system consisted of 10 *μ*L 2× SuperReal PreMix Plus, 1 *μ*L of each forward and reverse primer (10 pM each), 1 *μ*L of the template cDNA (80 ng), and 7 *μ*L of double-distilled water. The reaction was performed on a ABI7500 PCR system, and the reaction conditions were as follows: predenaturation at 95°C for 15 min, followed by 40 cycles of denaturation at 95°C for 10 s, and annealing and extension at 60°C for 32 s. Technical triplicates were prepared for each sample. The gene expression levels of the wild-type Th33 subjected to copper treatment were used as a reference, and the 2^−△△CT^ method was used for the calculation of gene expression levels in other samples. The internal reference gene was the* T. harzianum* glyceraldehyde 3-phosphate dehydrogenase (GAPDH) gene. Primers used in this study are shown in Table S1 in Supplementary Materials.

## 3. Results and Analysis

### 3.1. Comparison of Growth Rates between the* T. harzianum* Th33 Wild-Type Strain and the Mutant Strain Δthmea1

**Figure 1:**
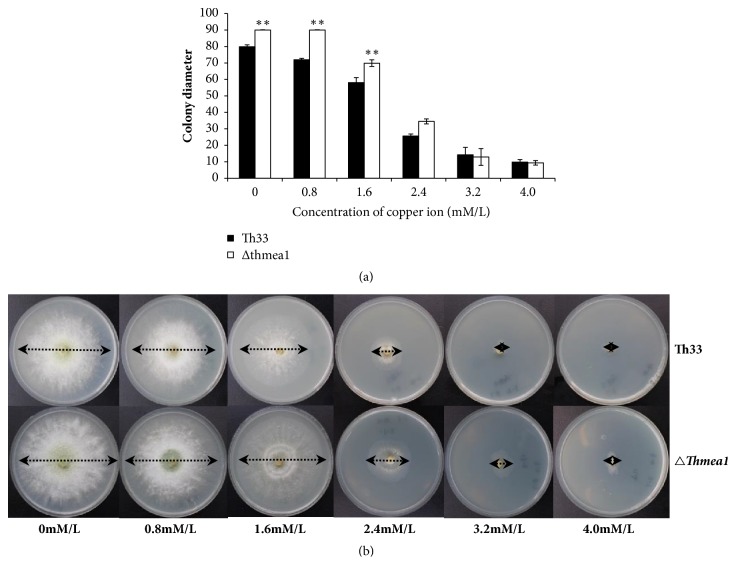
Effects of copper ions on the growth rate and colony morphology of* Trichoderma harzianum* Th33 and* thmea1* gene knockout mutant* Δthmea1*. (a) Colony diameter of* T. harzianum* Th33 wild-type and mutant* Δthmea1* under different copper iron concentrations at 28°C for 2 days. (b) Colony morphology of* T. harzianum* Th33 wild-type and mutant* Δthmea1* strains under different copper concentrations at 28°C for 2 days. Note: figure data is expressed as mean ± standard error. *∗* represents significance level of differences under different treatments (*∗* p<0.05; *∗∗* p<0.01).

The growth rate of the mutant* Δthmea1* in PDA culture media was higher than the wild-type strain, and copper ions imparted inhibitory effects on the growth of both strains. The colony diameters of the wild-type Th33 and the mutant* Δthmea1* strains grown on PDA plates with copper concentrations of 0 mM, 0.8 mM, 1.6 mM, 2.4 mM, 3.2 mM, and 4.0 mM after culturing for 2 days at 28°C were showed in Figure 1(a). With increasing copper concentrations, the growth rate of the colonies decreased. Within the copper concentration range of 0-2.4 mM, the growth rate of* Δthmea1* was significantly faster than wild-type Th33. However, at copper iron concentrations of 3.2 mM and 4.0 mM, the growth of both strains was severely inhibited, without significant differences. Under low copper concentrations,* Δthmea1* exhibited a higher colony growth rate and greater amount of aerial hypha compared to the wild-type strain after 2 days of growth, as shown in Figure 1(b). The formula for calculating the growth inhibition rate of the wild-type Th33 was y = 0.2755x + 0.0316, r = 0.969, in which y was the growth inhibition rate of the colony and x was the copper concentration. The regression formula for growth inhibition rate of* Δthmea1* was y = 0.282x − 0.0409, r = 0.976. The MIC_50_ of the wild-type Th33 strain and* Δthmea1* was 1.70 mM and 1.92 mM, respectively. The copper tolerance of* Δthmea1* showed a 12.9% increase compared to Th33, showing that* Δthmea1* had a significantly higher copper tolerance than the wild-type strain.

### 3.2. Total RNA Extraction and Construction of Sequencing Libraries

The samples used for transcriptome sequencing were hyphae from wild-type Th33 that was grown in liquid culture containing 0.8 mM copper irons (Th33-0.8-1 and Th33-0.8-2) and hyphae from* Δthmea1* that was grown in liquid culture containing 0.8 mM copper irons (*ΔTH-0.8-1* and* ΔTH-0.8-2*). Two biological replicates were prepared for one strain, generating a total of four samples. The electropherogram of the total RNA of the 4 samples showed clear separate bands of 18S and 28S rRNA, whereas that of the 5S band was weaker, indicating low RNA degradation and thus the RNAs were intact (figure not shown). The RIN values of the samples were ≥9.8 and quality evaluation was Grade I. Therefore, these qualified RNA samples were used for library construction.

### 3.3. Quality Evaluation of the Raw Data

Transcriptome sequencing was conducted on an Illumina HiSeq 2500 platform. The total yields of the four samples were 2,695,311,250 bp, 2,803,014,250 bp, 3,137,172,000 bp, and 2,952,492,750 bp, respectively, and the base contents were all > 2.6 Gb. For base quality scores, bases with a Phred score of Q20 (%) were all ≥92%. Therefore, the sequencing quality of this experiment was satisfactory and thus can be used for subsequent quantitative analysis of expression levels.

### 3.4. Gene Annotation and Quantitative Analysis of Expression Levels

The* T. harzianum* Th33 genome sequence was used as reference, and Tophat 2.0.12 software was used for alignment of sequencing reads. The proportion of reads from various samples that were aligned and mapped to the genome was > 80% (Table S2). Quantitation of gene expression was conducted using the FPKM method. The results of analysis of 10,849 genes showed that the proportion of genes that had a gene expression abundance of 10–50 was the highest, accounting for 31% of the total number of genes (Table S3).

### 3.5. Analysis of DEGs

**Figure 2:**
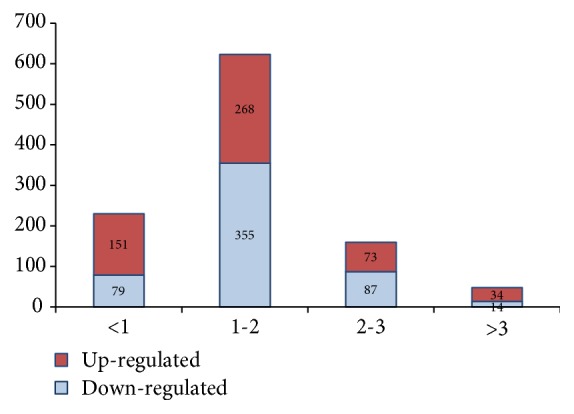
Differentially expressed genes of ΔTH-0.8_VS_Th33-0.8.

Cufflinks were used for analysis of DEGs between samples (*P* ≤0.01). Under copper stress, a total of 1,061 DEGs were identified between the knockout mutant* Δthmea1* and wild-type Th33, of which 526 genes were upregulated and 535 genes were downregulated. Statistical analysis indicated that for differential expression genes with a one- to twofold absolute value of log_2_(FC), there were 268 upregulated genes and 355 downregulated genes; for genes with a two- to threefold log_2_(FC) value, there were 73 upregulated genes and 87 downregulated genes; for genes with a more than threefold log_2_(FC) value, there were 34 upregulated genes and 14 downregulated genes. The results showed that DEGs with a one-to twofold log_2_(FC) accounted for a higher proportion, as shown in Table S4 and Figure 2.

### 3.6. GO Enrichment Analysis of DEGs

**Figure 3:**
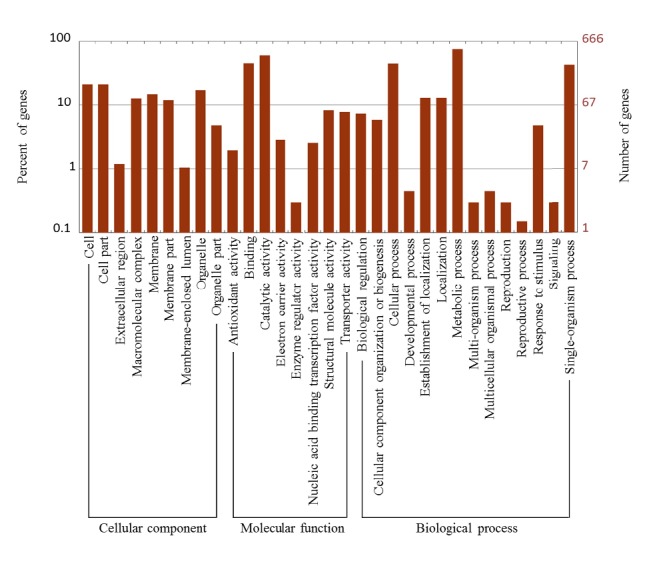
GO enrichment analysis of differentially expressed genes.

Blast2GO [7, 10] program was used for GO analysis. The program extracted the GO terms associated with the homologies identified by BLAST and returned a list of GO annotations, which were presented as hierarchical categories of increasing specificity. GO enrichment analyses were performed using Fisher's exact test with multiple testing corrections and an FDR of 0.05. A total of 666 DEGs were categorized into 31 functional groups in three main categories, “cellular component,” “molecular function,” and “biological process” (Table S5 and Figure 3). Some unigenes were assigned to multiple categories of GO terms, whereas others could not be assigned to a given GO term. In the cell component category, “cell” (113, 16.97%), “cellular components” (113, 16.97%), and “organelle” (92, 13.81%) were the most abundant terms. In the molecular function category, genes associated with “binding” (145, 21.77%) and “catalysis” (246, 36.94%) were the most abundant terms. In the biological process category, “cellular processes” (250, 37.54%), “metabolic processes” (451, 67.72%), and “single-organism process” (233, 34.98%) were the most abundant terms, as shown in Table S5 and Figure 3.

### 3.7. KEGG Pathway Enrichment Analysis of DEGs

**Figure 4:**
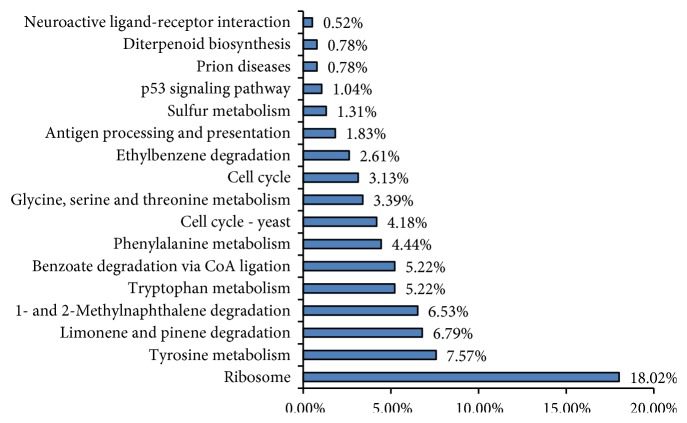
Pathway enrichment analysis of DEGs. The percentage of differentially expressed genes involved in KEGG pathways. Only the top 17 most abundant KEGG pathways are represented.

KEGG pathway analysis annotated 338 DEGs to 191 metabolic pathways.  Table S6 and Figure 4 showed the top 17 metabolic pathways that showed relatively high enrichment significance (P≤0.1). Among these pathways, ribosomal protein synthesis-associated pathways and genes accounted for 18.02% of the DEGs, genes involved in amino acid metabolism accounted for 20.62% (tryptophan metabolism; phenylalanine metabolism; glycine, serine and threonine metabolism, and tyrosine metabolism), and genes associated with cell cycle accounted for 7.31% (cell cycle, and cell cycle-yeast).

### 3.8. Genes Associated with Antioxidant Enzymes

The expression of antioxidant enzymes in the mutant* Δthmea1* and wild-type Th33 strains including catalase (Tha_07751, CAT), five peroxidases (Tha_04316, Tha_00485, Tha_00674, Tha_02847, and Tha_10717, POD), and copper and zinc superoxide dismutase genes (Tha_04932, SOD) was analyzed. Among these genes, Tha_07751, Tha_10717, and Tha_02847 were downregulated, whereas Tha_04316 was upregulated. This showed that when* Δthmea1* encountered copper stress, the expression levels of some antioxidant enzymes underwent varying degrees of up- or downregulation. In addition, analysis of genes associated with glutathione (GSH) showed that there were three out of four glutathione S-transferase (GST) genes in the mutant* Δthmea1* strain (Tha_03629, Tha_05984, and Tha_10746) and glutathione peroxidase (Tha_06359) that were upregulated, whereas one GST gene (Tha_01711) was downregulated (Table 1). This showed that the overall expression levels of genes associated with GSH enzymes in* Δthmea1* increased under copper stress.

### 3.9. Ribosomal Proteins and Heat Shock Protein- (HSP-) Related Genes

Ribosomal proteins are important components of ribosomes and play important roles in intracellular protein synthesis. HSPs have been reported to be generated under multiple stress conditions and participate in the repair and degradation of stress-damaged proteins as well as the folding, transportation, and assembly of newly synthesized peptide chains [11]. Under copper stress, there were 65 genes associated with ribosomal proteins in the mutant* Δthmea1* that showed differential expression, of which 60 genes were upregulated. In addition, the FPKM values of these genes were around twofold higher than that in the wild-type fungi, which were around 1,100–4,300 (Table S6). Under copper stress, the expression levels of 12 HSPs in the mutant* Δthmea1* were higher than the wild-type strain. Among these genes, Tha_00375, Tha_03314, Tha_09210, Tha_10420, and Tha_10740 showed significant expression (Table 2). These results suggested that the loss of the* thmea1* gene increased the synthesis capacity of ribosomal proteins and HSPs in* Δthmea1* under copper stress, thereby ensuring protein synthesis, supplementation, and repair.

### 3.10. Genes Associated with Copper Metabolism

Copper metabolism mainly includes uptake, intracellular storage and utilization, and extracellular release of copper ions. The expression of copper metabolism associated genes under copper stress were analyzed and showed in Table 3. Under copper stress, the copper reductase gene Tha_07043 (Fre2) was significantly upregulated in the* Δthmea1* mutant, but both strains showed relatively low expression levels. Among genes encoding for the four copper transporter proteins (Ctrp), Tha_02284 was significantly downregulated, but the FPKM values of the two strains were relatively low, whereas there were no significant differences in expression levels in Tha_01417, Tha_03591, and Tha_05828 between the two strains. This suggested that there may be no significant difference in the uptake of copper ions between the two strains. The genes for the copper chaperone proteins Tha_01680 (Ccs1) and Tha_07893 (Cox17) were not differentially expressed in the wild-type and* Δthmea1* strains, whereas the genes for the copper chaperone protein Tha_00261 (Atx1) and the P-type ATPase Tha_08191 (Ccc2) were upregulated in* Δthmea1*. This indicated that there were more copper ions entering the Golgi vesicles in the mutant strain. In addition, in the two strains, the multicopper oxidase gene Tha_02340 (Fet3) that participates in utilization and secretion of copper ions in Golgi vesicles was expressed at low transcriptional level. However, the copper amine oxidase gene Tha_03788 showed upregulated expression in the knockout mutant* Δthmea1*. These findings suggested that Tha_03788 may have a similar function to the multicopper oxidase gene Fet3. The aforementioned results showed that, under copper stress, the knockout mutant* Δthmea1* may exhibit stronger secretion and release of intracellular copper ions, thereby increasing its tolerance to copper ions. The metallothionein gene Tha_07074 (MT) had higher expression levels in both strains but no significant differences in expression levels between wild Th33 and* Δthmea1*, suggesting that this protein may play an important role in the release of copper ions in cells and was not regulated by Thmea1.

### 3.11. Validation of Transcriptome Sequencing Results

**Figure 5:**
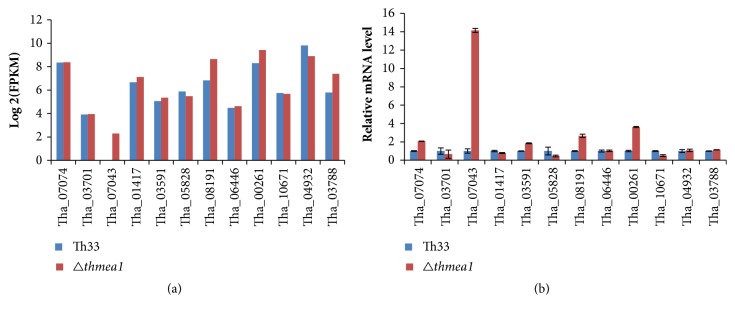
Validation of transcriptome sequencing results. (a) The transcriptome expression levels of 12 differentially expressed genes were expressed as log_2_FPKM; (b) qRT-PCR analysis of 12 differentially expressed genes. Note: figure data is expressed as the mean ± standard error.

To validate the reliability of transcriptome sequencing results, we selected 12 DEGs for qRT-PCR analysis. These DEGs included the metallothionein gene (Tha_07074), mac1 transcription factor (Tha_03701), iron reductase (Tha_07043), the genes for three transporter proteins, (Tha_01417, Tha_03591, and Tha_05828), the genes for two P-type ATPases (Tha_08191, Tha_06446), the copper chaperone gene (Tha_00261), genes for two superoxide dismutase (Tha_10671, Tha_04932), and the copper amine oxidase gene (Tha_03788). The melting curves of each gene were analyzed by qRT-PCR, and a single peak was observed for each gene product (Figure S1). qRT-PCR analysis showed that the expression levels and trends of these DEGs coincide with transcriptome sequencing results (log_2_FPKM), as shown in Figure 5.

## 4. Discussion

To detect changes in copper tolerance in the* T. harzianum* Th33* thmea1* mutant* Δthmea1*, transcriptome sequencing of the* T. harzianum* Th33 wild-type and mutant* Δthmea1* strains under copper stress was performed. The* T. harzianum* GAPDH coding gene* gapdh* was used as internal reference for qRT-PCR validation of 12 selected* T. harzianum* genes. The validation results coincided with the transcriptome sequencing results, thereby demonstrating the reliability of our findings. The expression levels of the antioxidant enzymes, glutathione-related enzymes, ribosomal proteins, HSPs, and copper metabolism associated genes in wild-type Th33 and* Δthmea1* were analyzed to examine the function of the* thmea1* gene in this study.

Copper stress causes the accumulation of large amounts of reactive oxygen species in cells [11, 12]. Generally, it is believed that cells activate and increase the levels of antioxidant enzymes such as CAT, POD, and SOD and reduce that of glutathione (GSH) to alleviate oxidative stress [13]. GST is a key enzyme that catalyzes the binding of glutathione and metal ions and plays an important role during the binding of glutathione to copper ions [14]. In addition, glutathione peroxidase has functions such as reducing toxic peroxide compounds and protecting the structure and function of the cell membrane [15, 16]. In this study, the expression of antioxidant enzymes in* Δthmea1* showed varying degrees of up- and downregulation under copper stress, whereas GST showed a general upregulated expression. These findings suggested that* Δthmea1* changed the activity of antioxidant enzymes and increases the expression levels of GST to increase the copper-binding capacity and ability to clear toxic peroxide compounds, thereby alleviating the damage caused by reactive oxygen species to cells.

Ribosomes are major sites in the cell where proteins are synthesized and ribosomal proteins are important components of ribosomes [17]. HSPs participate in the repair and degradation of stress-damaged proteins, as well as the folding, transportation, and assembly of newly synthesized peptide chains [18]. These two types of proteins play important roles during protein synthesis. Under copper stress, the expression of eight HSP genes and 60 ribosomal protein genes in the mutant* Δthmea1* was upregulated. These findings suggested that the loss of the* thmea1* gene in* T. harzianum* resulted in an increase in protein synthesis and repair capabilities.

Bioinformatics analysis showed that the amino acids of Thmea1 contained 3 conserved C2H2 domains, which were identical to C2H2 transcription factors Swi5 and Ace2 in* Saccharomyces cerevisiae*. Swi5 was an activator of HO gene transcription, and Ace2 was a metallothionein expression activator; Swi5 and Ace2 had functional similarities [19, 20]. Metallothioneins (MTs) are rich in cysteine and can effectively bind Cu^+^ ions to decrease free copper ions and alleviate heavy metal stress [21]. This had been observed in the yeast strain with tandem expression of the yeast MT gene, Cup1, which could tolerate 12 mM of copper ions, whereas normal strains could only tolerate 1.75 nM/L of copper ions [22]. In this study, the MT gene (Tha_07074) had higher expression levels in both wild Th33 and the* Δthmea1*, but there were no significant differences between the two strains, indicating that MT protein played an important role in responding to copper stress and was not affected by* thmea1* in* T. harzianum. *Thmea1 did not show the same function as Swi5 and Ace2.

**Figure 6:**
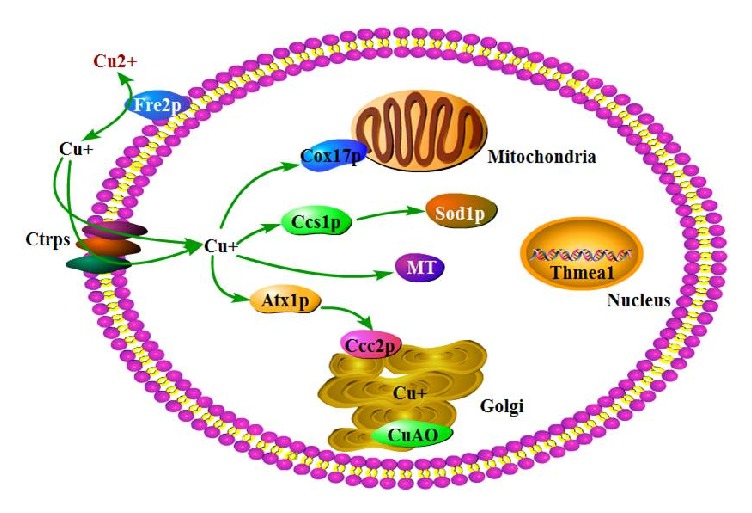
Proposed copper metabolism diagram in* Trichoderma harzianum*.

Organisms have developed a set of strict and relatively conserved protective mechanisms to maintain copper homeostasis when encountering copper stress. Research on copper metabolism mechanisms in yeast was relatively clearer [23–25], which included the uptake, storage, mitigation, and secretion of copper ions. Using yeast metabolism as reference, we analyzed genes associated with copper metabolism in wild-type* T. harzianum* Th33 and the mutant* Δthmea1* under copper stress. In yeast, copper ions were reduced by the copper and iron reductase Fre1p/Fre2p on the plasma membrane into Cu^+^ ions before uptake. Subsequently, these ions were captured by the high-affinity copper transporter protein Ctr1p/Ctr3p or the low-affinity copper transporter protein Ctr2p and transported into cells [26, 27]. The present study determined that, under copper stress, the copper reductase gene Tha_07043 (Fre2) was significantly upregulated in the* Δthmea1* mutant, but both strains showed relatively low expression levels. This finding suggested that the intake of copper ions in both strains was relatively low. Similarly, the gene expression levels of four copper transporter proteins (Ctrp), namely, Tha_01417, Tha_02284, Tha_03591, and Tha_05828, in both strains were relatively low. This showed that* thmea1* may not have a strong correlation with intracellular transport of copper ions. However, whether there are other copper transportation channels present in* Trichoderma* requires further study. In yeast, Cu^+^ ions that enter the cell were mainly bound by three copper chaperone proteins and transported to corresponding target molecules and organelles. The copper chaperone protein Ccs1p transported coppers to copper and zinc superoxide dismutase Sod1p to remove intracellular reactive oxygen species and alleviate oxidative stress in cells [28, 29]. Cox17 was responsible for transporting copper ions to the mitochondria and SCO2 mediated the transport of copper ions into the cytochrome c oxidase pathway [30]. Atx1p transported bound copper ions to the Golgi bodies, which then transported Cu^+^ to the Golgi vesicular system by the P-type ATPase Ccc2p that was located on the Golgi bodies [31, 32]. Finally, these ions were utilized in the synthesis of the multicopper oxidase Fet3p and secreted along with this protein [33]. This process played a crucial role in regulating intracellular copper homeostasis [34]. Our study found that the copper chaperone genes Tha_01680 (Ccs1) and Tha_07893 (Cox17) were not differentially expressed in the wild-type and* Δthmea1* strains under copper stress, but both of them were expressed at low transcriptional level, whereas the copper and zinc superoxide dismutase gene Tha_04932 (Sod1) was upregulated in both strains, with the wild-type Th33 strain showing higher levels than the mutant strain. Therefore, we hypothesized that the* T. harzianum* copper chaperone proteins Ccs1p and Cox17p were not regulated by* thmea1*. However, Sod1p could clear reactive oxygen species in cells, thereby suggesting that* thmea1* may have some role in the regulation of superoxide dismutase expression. The copper chaperone gene Tha_00261 (Atx1) and the P-type ATPase gene Tha_08191 (Ccc2) showed upregulated expression in the mutant* Δthmea1*. It was possible that the uptake rate of copper ions into the Golgi vesicular secretion system of the mutant strain* Δthmea1* was relatively higher, and there were no changes in the transcript levels of the multicopper oxidase gene Tha_02340 (Fet3), which was responsible for the secretion of copper ions. However, the expression levels of the copper amine oxidase gene Tha_03788 (CuAO) increased in the knockout mutant* Δthmea1*. We speculated that knocking out the* thmea1* gene increased the ability of* T. harzianum* to secrete intracellular copper ions and Tha_03788 might (CuAO) have a similar function to the multicopper oxidase gene Fet3 in* T. harzianum* and required further validation. Based on the above results and the findings of De Freitas et al. [23] on yeast copper metabolism, we described the copper metabolic diagram of* T. harzianum* (Figure 6). We hypothesized that after* T. harzianum* lost its* thmea1* gene, the ability of cells to scavenge reactive oxygen species, mainly through the glutathione antioxidant system, was enhanced, whereas protein synthesis and repair and copper secretion increased under copper stress, which increased the ability of the mutant strain to tolerate copper stress.

## 5. Conclusions

The C2H2 transcription factor gene* thmea1* is a negative regulated factor of copper tolerance ability in* T. harzianum* and does not show metallothionein expression activator activities. Lacking of* thmea1* increases the copper tolerance of* T. harzianum*, and the ability of* Δthmea1 *to scavenge reactive oxygen species, mainly through the glutathione antioxidant system, is enhanced, protein synthesis and repair ability are enhanced by upregulating the expression of hot shock proteins and ribosomal proteins, and the secretion ability of copper irons is enhanced by upregulating the expression of copper chaperone protein Tha_00261 (Atx1), the P-type ATPase Tha_08191 (Ccc2), and copper amine oxidase gene Tha_03788 (CuAO), which increases the ability of the mutant strain to tolerate copper stress.

## Figures and Tables

**Table 1:** Expression of genes associated with antioxidant enzymes under copper stress.

Gene ID	Th33-0.8 FPKM	*ΔTH-0.8* FPKM	log_2_ (FC)	P_value	Annotation
Tha_07751	258.9260	99.9329	-1.3735	0.00005	Catalase
Tha_04316	62.6029	195.1620	1.6404	0.00005	Peroxidase
Tha_00485	216.0380	431.4815	0.9980	0.00005	Peroxidase
Tha_00674	293.2600	519.5290	0.8250	0.00005	Peroxidase
Tha_02847	256.7990	124.8930	-1.0399	0.00005	Peroxidase
Tha_10717	165.2110	40.6521	-2.0229	0.00005	Peroxidase
Tha_04932	897.1265	477.6965	-0.9092	0.00005	Copper, zinc superoxide dismutase
Tha_03629	6.9580	38.1848	2.4563	0.00005	Glutathione S- transferase
Tha_05984	35.1119	188.6655	2.4258	0.00005	Glutathione S- transferase
Tha_10746	141.4985	613.1045	2.1154	0.00005	Glutathione S- transferase
Tha_06359	211.2125	502.8465	1.2514	0.00005	Glutathione peroxidase
Tha_01711	72.0724	31.9052	-1.1757	0.00005	Glutathione S- transferase

log_2_(FC) values of p value < 0.01 and |log2 (FC)|≥1.0 were considered to be statistically significant.

**Table 2:** Expression of genes associated with heat shock proteins under copper stress.

Gene ID	Th33-0.8 FPKM	ΔTH-0.8 FPKM	log_2_ (FC)	P_value	Annotation
Tha_00375	3928.4350	17740.9000	2.1751	0.00005	HSP 30
Tha_01588	39.5399	72.1441	0.8676	0.0001	Endoplasmic reticulum HSP
Tha_03314	1349.0900	4187.4700	1.6341	0.00005	HSP 70
Tha_04863	329.5615	572.9580	0.7979	0.00005	HSP 101
Tha_05072	99.0282	180.4830	0.8660	0.00005	HSP 70
Tha_09210	50.5491	148.5090	1.5548	0.00005	HSP 78
Tha_10420	2218.2100	8353.4400	1.9130	0.00005	Small HSP
Tha_10740	446.6465	1410.3950	1.6589	0.00005	HSP 80
Tha_03207	83.88795	201.6835	1.26556	0.00005	hsp70 family protein, mitochondrial precursor
Tha_07533	96.4268	186.609	0.95251	0.00005	hsp60 mitochondrial precursor-like protein
Tha_01643	95.8451	46.79905	-1.03422	0.00005	hsp70-like protein
Tha_00917	414.4935	883.909	1.09255	0.00005	hsp70 family protein

log_2_(FC) values of p value < 0.01 and |log2 (FC)|≥1.0 were considered to be statistically significant.

**Table 3:** Expression of genes associated with copper metabolism under copper stress.

Gene ID	Th33-0.8 FPKM	ΔTH-0.8 FPKM	log_2_ (FC)	P_value	Annotation
Tha_07043	0.5825	4.8977	3.0718	0.00005	iron reductase Fre2
Tha_02284	18.6548	7.2156	-1.3703	0.00075	copper transporter protein Ctrp
Tha_01417	102.2066	138.307	0.4364	0.12985	copper transporter protein Ctrp
Tha_03591	33.4524	40.7149	0.2834	0.2423	copper transporter protein Ctrp
Tha_05828	59.133	44.4831	-0.4107	0.0701	copper transporter protein Ctrp
Tha_01680	54.4918	51.1831	-0.09037	0.394517	copper chaperone protein Ccs1
Tha_07893	83.8104	90.8476	0.1163	0.286482	copper chaperone protein Cox17
Tha_00261	313.5735	687.164	1.1318	0.00005	copper chaperone protein Atx1
Tha_08191	113.641	400.6475	1.8178	0.00005	P-type ATPases Ccc2
Tha_02340	0.3347	0.4288	0.3574	0.418922	multi-copper oxidase gene Fet3
Tha_03788	55.4367	167.685	1.5968	0.00005	copper amine oxidase CuAO
Tha_07074	325.4160	332.0320	-0.0290	0.91225	Metallothionein MT

log_2_(FC) values of p value < 0.01 and |log2 (FC)|≥1.0 were considered to be statistically significant.

## Data Availability

The data used to support the findings of this study are included within the article.

## Supplementary Materials

Supplementary Materials The supplementary material contains 7 tables and 1 figure. Table S1: primers used in validation experiments. Table S2: mapping rate of sequencing samples of Th33 and* Δthmea1*. Table S3: gene number of different expression level (FPKM) distribution. Table S4: list of differentially expressed genes (DEGs) showed in Figure 2. Table S5: number of DEGs assigned to GO classification. Table S6: summary of DEGs assigned to the KEGG database. Table S7: list of 65 ribosomal protein genes with significantly altered expression in* T. harzianum*. Figure S1: the melting curve of the tested genes analyzed by qRT-PCR.
