# Supplementary material for: Functions of the C2H2 Transcription Factor Gene thmea1 in Trichoderma harzianum under Copper Stress Based on Transcriptome Analysis
Source: Biomed Res Int. 2018 Jul 18;2018:8149682. doi: 10.1155/2018/8149682 (PMC6076916; doi:10.1155/2018/8149682)
Supplement: Supplementary Materials — The supplementary material contains 7 tables and 1 figure. Table S1: primers used in validation experiments. Table S2: mapping rate of sequencing samples of Th33 and Δthmea1. Table S3: gene number of different expression level (FPKM) distribution. Table S4: list of differentially expressed genes (DEGs) showed in Figure 2. Table S5: number of DEGs assigned to GO classification. Table S6: summary of DEGs assigned to the KEGG database. Table S7: list of 65 ribosomal protein genes with significantly altered expression in T. harzianum. Figure S1: the melting curve of the tested genes analyzed by qRT-PCR. [file 8149682.f1.zip › Figure S1_BMRI_2366629.doc]

**Tha_07074**

**Melt curve**

**Tha_03701**

**Melt curve**

**Tha_07043**

**Melt curve**

**Tha_03591**

**Tha_01417**

**Melt curve**

**Tha_05828**

**Tha_08191**

**Tha_06446**

**Tha_00261**

**Tha_10671**

**Tha_04932**

**Tha_03788**

Figure S1: the melting curves of tested genes analyzed by qRT-PCR
